# Carboxy-Terminal Cementum Protein 1-Derived Peptide 4 (cemp1-p4) Promotes Mineralization through wnt/*β*-catenin Signaling in Human Oral Mucosa Stem Cells

**DOI:** 10.3390/ijms21041307

**Published:** 2020-02-15

**Authors:** Rita Arroyo, Sonia López, Enrique Romo, Gonzalo Montoya, Lía Hoz, Claudia Pedraza, Yonathan Garfias, Higinio Arzate

**Affiliations:** 1 Laboratorio de Biología Periodontal, Facultad de Odontología, Universidad Nacional Autónoma de México, CDMX 04510, Mexico; rittaarroyo@yahoo.com.mx (R.A.); sonyletayf@hotmail.com (S.L.); dr_roaren@yahoo.com.mx (E.R.); jimago_jar@hotmail.com (G.M.); lahry@hotmail.com (L.H.); patpedrazamora@gmail.com (C.P.); 2 Departamento de Bioquímica, Facultad de Medicina, UNAM, Universidad Nacional Autónoma de México, CDMX 04510, Mexico; ygarfias@bq.unam.mx; 3 Instituto de Oftalmología Conde de Valenciana, CDMX 06800, Mexico

**Keywords:** cementum, peptide, *β*-catenin, cell differentiation, CEMP1-p4, mineralization, stem cells

## Abstract

Human cementum protein 1 (CEMP1) is known to induce cementoblast and osteoblast differentiation and alkaline phosphatase (ALP) activity in human periodontal ligament-derived cells in vitro and promotes bone regeneration in vivo. CEMP1′s secondary structure analysis shows that it has a random-coiled structure and is considered an Intrinsic Disordered Protein (IDP). CEMP1′s short peptide sequences mimic the biological capabilities of CEMP1. However, the role and mechanisms of CEMP1′s *C*-terminal-derived synthetic peptide (CEMP1-p4) in the canonical Wnt/*β*-catenin signaling pathway are yet to be described. Here we report that CEMP1-p4 promotes proliferation and differentiation of Human Oral Mucosa Stem Cells (HOMSCs) by activating the Wnt/*β*-catenin pathway. CEMP1-p4 stimulation upregulated the expression of *β*-catenin and glycogen synthase kinase 3 beta (GSK-3B) and activated the transcription factors TCF1/7 and Lymphoid Enhancer binding Factor 1 (LEF1) at the mRNA and protein levels. We found translocation of *β*-catenin to the nucleus in CEMP1-p4-treated cultures. The peptide also penetrates the cell membrane and aggregates around the cell nucleus. Analysis of CEMP1-p4 secondary structure revealed that it has a random-coiled structure. Its biological activities included the induction to nucleate hydroxyapatite crystals. In CEMP1-p4-treated HOMSCs, ALP activity and calcium deposits increased. Expression of Osterix (OSX), Runt-related transcription factor 2 (RUNX2), Integrin binding sialoproptein (IBSP) and osteocalcin (OCN) were upregulated. Altogether, these data show that CEMP1-p4 plays a direct role in the differentiation of HOMSCs to a “mineralizing-like” phenotype by activating the *β*-catenin signaling cascade.

## 1. Introduction

The periodontium is a unique, complex and functional biological system that provides support to the dental organs. It is composed of two connective mineralized tissues—alveolar bone and cementum and two soft connective tissues, gingiva and periodontal ligament. However, the commonly occurring disease, periodontitis, leads to loss of homeostasis and consequently to the destruction of the tooth-supporting structures [1]. Therefore, it is the aim of periodontal therapeutics to restore structurally and functionally the periodontium. For this to occur, a series of molecular events must be concerted timely and spatially. Of relevant importance for the regeneration of the periodontium is the formation of new cementum [2]. Nevertheless, the cellular and molecular mechanisms that regulate the formation of cementum are poorly understood [2]. This unique tissue covers the root surface and in conjunction with periodontal ligament and alveolar bone, provides a functional anchoring apparatus in the periodontium [3,4,5]. Recent evidence has shown that the lamina propria of the oral mucosa contains mesenchymal stem cell populations that can differentiate in order to form mineralized tissues [6]. A few years ago, cementum markers such as cementum protein 1 (CEMP1) and cementum attachment protein (CAP) were cloned and characterized [7,8]. These two proteins have shown to promote the proliferation and differentiation of periodontal ligament cells toward a “mineralizing-like” phenotype [9,10,11]. Preclinical studies have shown that CEMP1′s peptide sequences can be utilized in therapeutic interventions such as critical-sized calvarial defects, mimicking *hr*CEMP1′s (human recombinant Cementum Protein 1) biological and therapeutic capabilities [12]. Therefore, CEMP1′s short peptide sequences are possible regulators of stem cell differentiation toward a “mineralizing-like” cell phenotype [12]. 

The canonical Wnt/*β*-catenin signalling pathway is necessary for tooth development during epithelial-mesenchymal interactions and mineralized tissue formation, indicating that *β*-catenin plays a critical role in this signalling pathway [13,14,15]. The cumulative evidence has shown how the Wnt signalling pathway coordinates pluripotency and self-renewal of stem cells [16,17,18]. The Wnt signaling pathway stabilizes *β*-catenin, which binds to the T-cell factor/lymphoid enhancer-binding factor (TCF/LEF) transcription factors and mediates the transcription of Wnt target genes [19]. However, activated Wnt/*β*-catenin is not sufficient to induce osteogenic differentiation unless other stimulatory signals are present. In addition, little is known about the involvement of CEMP1′s peptide sequences on the canonical Wnt signaling pathway on cell differentiation to a “mineralizing-like” cell phenotype. Previous studies have shown that CEMP1′s N-terminal domain possesses biological properties to promote cell differentiation and mineralization. Analysis of CEMP1’s carboxy-terminus domain revealed that its secondary structure is 100% random-coiled and represents CEMP1′s most intrinsically disordered region (IDR). This polypeptide segment is likely to form a defined three-dimensional structure but is nevertheless functional. It is known that IDRs actively participate in diverse functions that increase the functional states in which a protein can exist in the cell. In this study, we aimed to determine whether CEMP1′s carboxy-terminus sequence triggers the Wnt signaling pathway in human oral mucosal stem cells (HOMSCs) to differentiate toward a “mineralizing-like” cell phenotype. 

## 2. Results

### 2.1. CEMP1-p4 Physicochemical Characteristics

**Figure 1:**
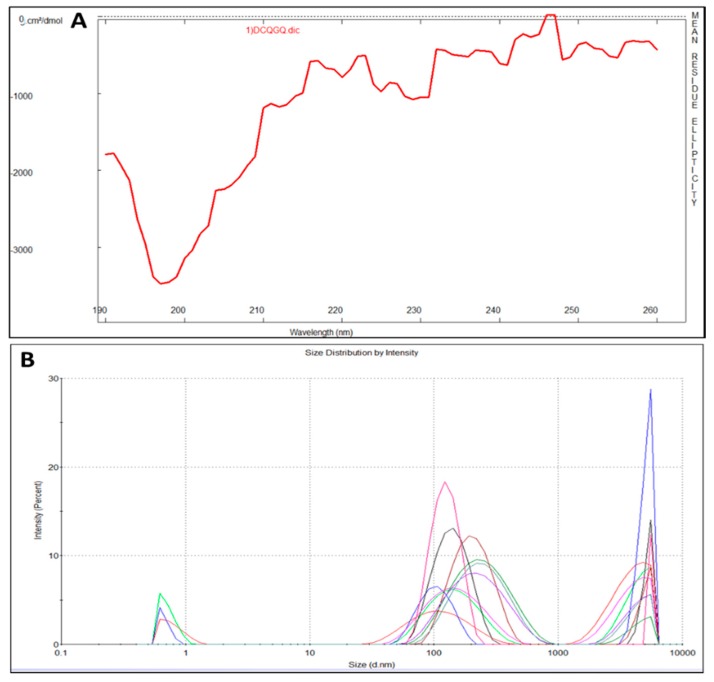
(**A**) Circular dichroism spectrum of cementum protein 1 *C*-terminal-derived synthetic peptide (CEMP1-p4). The lack of signals in the far UV region indicates that the peptide’s secondary structure is mainly random-coiled. (**B**) Dynamic light scattering measurements revealed the CEMP1-p4 peptide adopts three oligomerization states at 37 °C.

CEMP1-p4 has a molecular weight (MW) of 1595.72 Da with a theoretical isoelectric point of 4.03. Theoretical relative hydrophobicity is of 20.15 and GRAVY (grand average of hydropathy) is of −1.160. These characteristics make CEMP1-p4 a hydrophilic peptide. Circular dichroism of CEMP1-p1 showed a spectrum with negative weak signal values near to 197 nm. The spectrum reflects the random-coil CEMP1-p4 structure (Figure 1A). Dynamic light scattering analysis showed that CEMP1-p4 forms monomeric species of ~10 nm and polymeric aggregates of ~120 and ~5000 nm (Figure 1B).

### 2.2. CEMP1-p4 Induces Mineralization in a Cell-Free System

**Figure 2:**
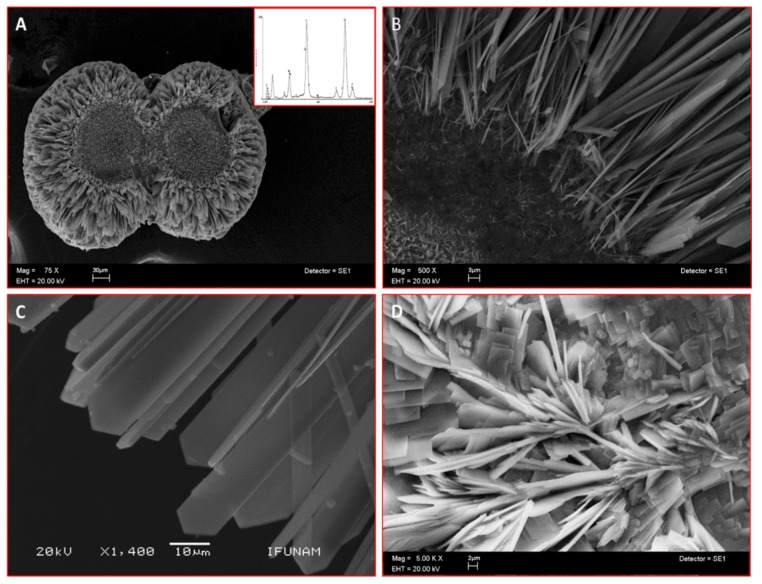
Scanning electron microscope (SEM) images of sphere-like structures formed in vitro by CEMP1-p4 (inset indicates Ca/P ratio of 1.52) (**A**). (**B**) Higher magnification shows an amorphous core with nascent needle-like crystals. (**C**) Hydroxyapatite crystals show a spear-like form. (**D**) Control crystals show irradiating crystals and plate-like crystals.

Scanning electron microscopy (SEM) analysis of the mineral formed in a semisolid gel in vitro by CEMP1-p4 revealed the formation of sphere-like structures with a size of ~225 µm (Figure 2A). These spheres contained a core of disorganized small crystals. This core seemed to give origin to nascent needle-like crystals, which showed a radially spatial arrangement (Figure 2B) and became densely packed and changed to a quadrangular feature, ending angularly at the growing front (Figure 2C). The Ca/P ratio of the analyzed crystals by energy-dispersive X-ray spectroscopy (EDS) was of 1.52 ± 0.03 (spectrum inset in 2A), like hydroxyapatite’s Ca/P ratio value in bone. Bovine serum albumin was used as a blank protein, which revealed that planar crystals were formed, and the deposited crystals resembled lamellae-like features. The crystals induced by BSA showed Ca/P ratio of 1.2 ± 0.06 (Figure 2D).

### 2.3. CEMP1-p4 Promotes Cell Proliferation

**Figure 3:**
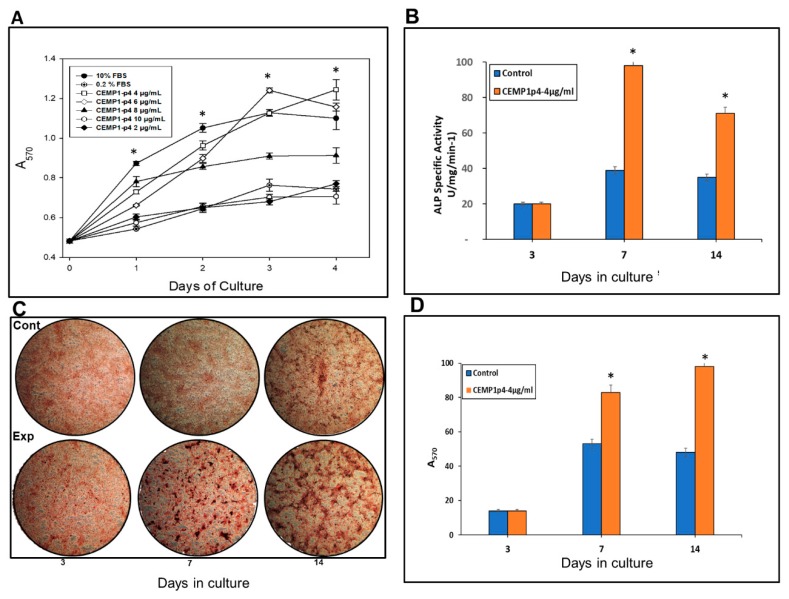
(**A**) Human oral mucosal stem cells (HOMSCs) were treated from 0 up to 96 h with several CEMP1-p4 concentrations where 4 µg/mL was shown to be optimal for cell proliferation. (**B**) CEMP1-p4 at 4 mg/mL increases alkaline phosphatase (ALP)-specific activity on HOMSCs at 7 and 14 days. (**C**) Representative images showing ARS after osteogenic induction on HOMSCs by CEMP1-p4 at 4 mg/mL during 3, 7, and 14 d of culture. (**D**) Mineral formation was quantified by cetylpyridinium chloride (CPC), showing that CEMP1-p4 is a potent inductor of calcium salts. *p < 0.05.

The cell proliferation assay demonstrated that CEMP1-p4 at a 4 µg/mL was the optimal concentration, since HOMSCs proliferated by 83%, 92%, 101% and 113% at 24, 48, 72 and 96 h respectively when compared with positive controls (Figure 3A).

### 2.4. CEMP1-p4 Stimulated ALP Specific Activity by HOMSCs

HOMSCs treated with CEMP1-p4 at 4 µg/mL increased alkaline phosphatase-specific activity at 7 and 14 days by 1.5 and 1.1-fold respectively, when compared to controls (Figure 3B).

### 2.5. CEMP1-p4 induced HOMSCS’s Mineralization

HOMSCs treated during 3, 7, and 14 days with CEMP1-p4 (4 µg/mL) showed that CEMP1-p4 induced HOMSCs to form mineralized calcium nodules. Control cells treated with 10% FBS supplemented with 5 mM *β*-glycerophosphate and 50 µg/mL ascorbic acid also formed calcium nodules (Figure 3C). However, mineral quantification demonstrated significant differences, since experimental cultures showed 58% and 107% increases on mineral deposits at 7 and 14 days of culture when compared to controls (Figure 3D). 

### 2.6. Effect of CEMP1-p4 on Cell Signaling Pathway

#### 2.6.1. CEMP1-p4 Induced mRNA Transcription of Wnt Signaling Pathway-Related Proteins

**Figure 4:**
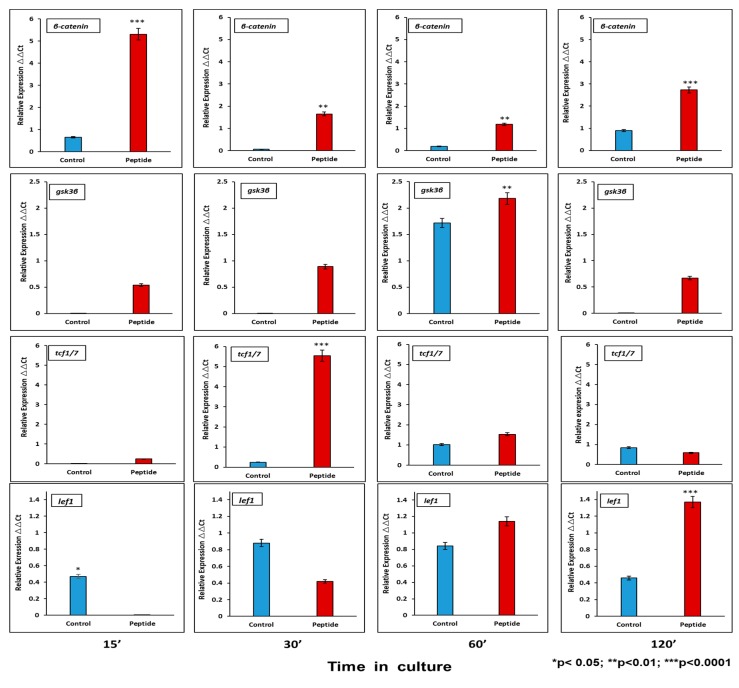
mRNA Wnt pathway signaling-related molecules analyzed at 15, 30, 60 and 120 min. CEMP1-p4 induced higher expression of *β*-catenin. At 60′ glycogen synthase kinase 3B (GSK3B) increased significantly. The transcription factor T-cell transcription factor (Tcf)/lymphoid enhancer binding factor 1 (Lef1) showed its higher expression at 30′ and 120´. Identical triplicates were prepared for each reaction, and the experiment was repeated 3 times independently (*n* = 3/group).

Activation of the canonical Wnt pathway has been described to affect proliferation and cell differentiation. At the mRNA level, CEMP1-p4 induces *β*-catenin relative expression by HOMSCs as early as 15 min after the addition of CEMP1-p4 by 6.6-fold, decreases at 30 and 60 min and increases by 2.1-fold at 2 h when compared to controls. Lef1 increases its expression by 2-fold at 30 min and Tcf1 increases by 18-fold at 30 min as compared to controls. GSK3b reaches its highest expression at 60 min by 1.2-fold when compared to controls (Figure 4). 

#### 2.6.2. CEMP1-p4 Affected Protein Levels of Wnt Signaling-Related Proteins

**Figure 5:**
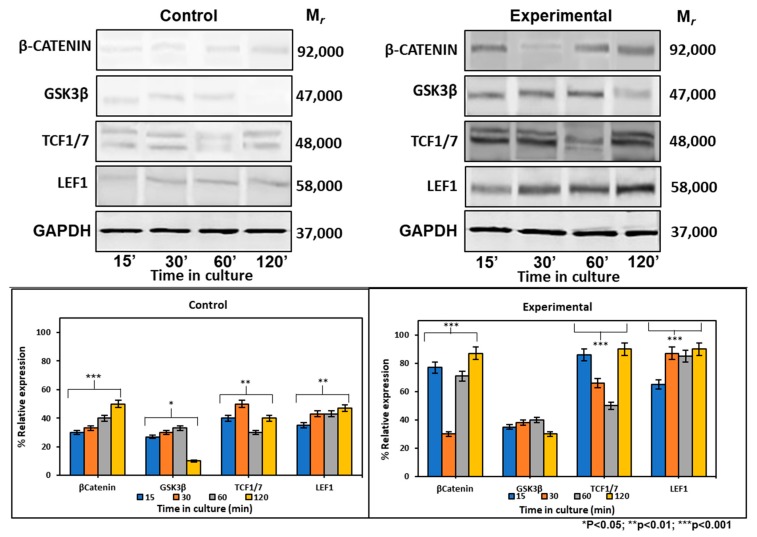
Expression of cell signaling pathway Wnt/*β*-catenin-related markers at the protein level. Control cultures showed that *β*-catenin, GSK, TCF1/7 and LEF1 showed low expression at 15, 30, 60 and 120 min of culture. HOMSCs cultures treated with CEMP1-p4 at 4 µg/mL increased β-catenin, TCF1/7 and LEF1 expression at 15, 30, 60 and 120 min respectively.

At the protein level, the expression of *β*-catenin increased by 2-, 0.7- and 0.7-fold at 15, 30, and 120 min respectively, after CEMP1-p4 addition. There were no GSK3*β* expression differences between experimental and control cultures. Tcf1/7 showed its highest expression at 15 and 120 min by 1- and 1.2-fold when compared to controls. Lef1 maintained a constant expression at all times tested and increased by 1-fold when compared to controls (Figure 5). These data taken together indicate that CEMP1-p4 induces the signaling pathway of Wnt/*β*-catenin.

### 2.7. Expression of Mineralization-Related Markers at the mRNA and Protein Levels

**Figure 6:**
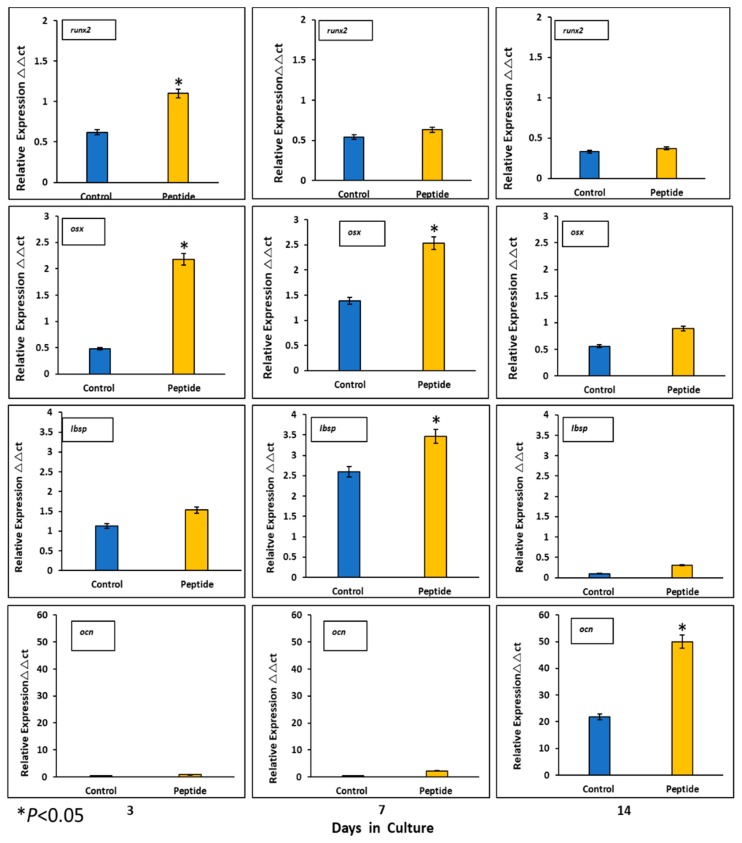
Expression of mineralization-related markers at the mRNA level. CEMP-1-p4 induced higher expression of core-binding factor subunit alpha-1 (RUNX2) and osterix (OSX) on days 3 and 7. Integrin-binding sialoprotein (IBSP) was highly expressed at the middle phase and osteocalcin (OCN) at the late stages of mineralization, when compared to controls. Identical triplicates were prepared for each reaction, and the experiment was repeated 3 times independently (*n* = 3/group). *p < 0.05.

Expression of mineralization-related markers at the mRNA level by HOMSCs at 3, 7 and 14 days revealed that at the initial stages of mineralization, RUNX2 and OSX increased by 2.1- and 3.7-fold and IBSP was barely expressed by 0.2-fold when compared to controls. At the middle stages of mineralization, OSX and IBSP increased its expression by 3.7- and 4.2-fold respectively, when compared to controls. OCN reached its maximum expression at late stages of mineralization by 1.7-fold when compared to controls. The results show that CEMP1-p4 promotes HOMSCs differentiation to a “mineralizing-like” cell phenotype (Figure 6).

**Figure 7:**
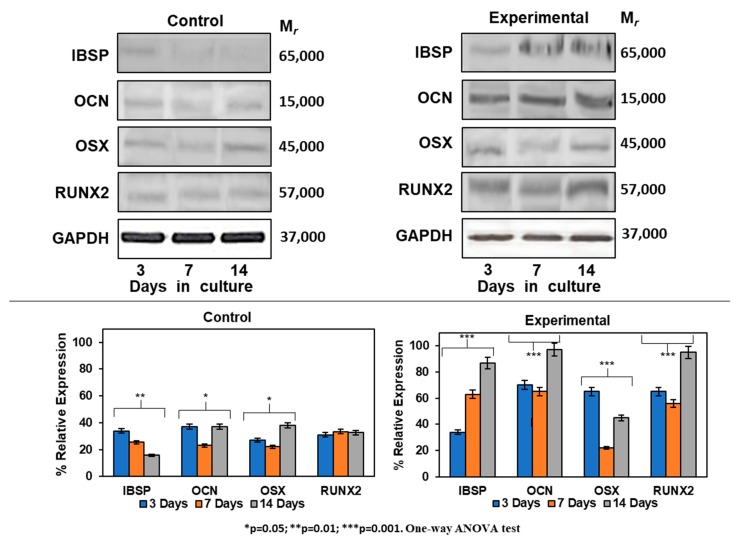
Expression of mineralization-related markers at the protein level. Experimental cultures treated with 4 µg/mL showed a higher expression of IBSP, OSX, RUNX2 and OCN at all terms tested.

The differentiation process of HOMSCs treated with CEMP1-p4 to a mineralizing phenotype was assessed by Western blot. Expression of IBSP increased by 0.4-, 1.6- and 5.0-fold at 3, 7 and 14 days, respectively, when compared to controls. Experimental cultures showed the increase of OCN by 2.4-, 2.2- and 3.3-fold as compared to controls at all terms tested. RUNX2 was expressed by 2.2-, 1.9- and 3.2-fold at 3, 7 and 14 days, respectively, when compared to controls. The expression of OSX revealed a positive effect of CEMP1-p4 on HOMSCs, since the expression values at 3, 7 and 14 days of culture were 2.2, 0.7 and 1.6, respectively, when compared to the controls (Figure 7). Taken together, these data show that HOMSCs treated with CEMP1-p4 express osteoprogenitor markers and differentiate to a “mineralizing-like” cell phenotype.

### 2.8. CEMP1-p4 was Internalized by HOMSCs 

**Figure 8:**
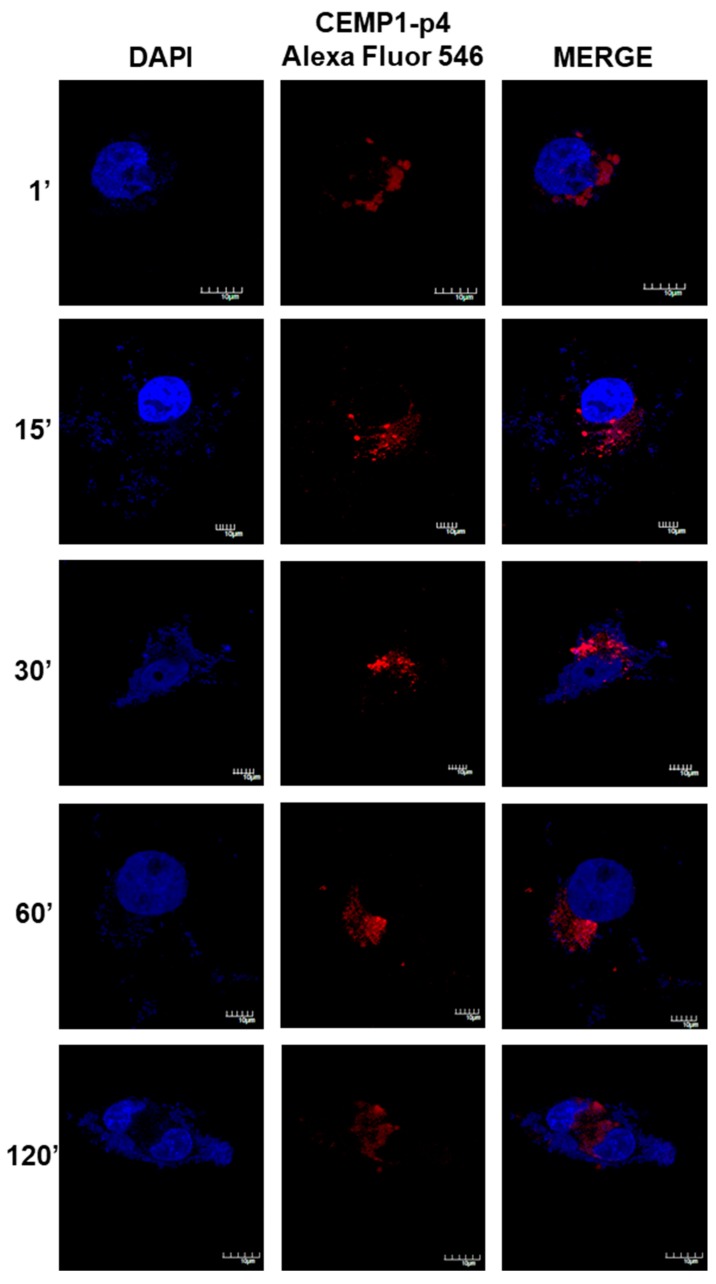
Confocal Microscopy Location of CEMP1-p4. CEMP-1-p4 labelled with Alexa Fluor 546 was mainly located surrounding the nucleus from the first minute and the pattern was diffuse and organized into granules and dispersed at 120 min. Original magnification, 3400. Scale bar = 10 μm.

After 1 min, HOMSCs treated with CEMP1-p4 labelled with Alexa Fluor 546 showed spontaneous internalization with a nuclear and mostly perinuclear localization, with a compact and homogeneous distribution. After 1 and 15 min of incubation, CEMP1-p4 aggregates in the cytoplasm **and** maintained nuclear and perinuclear location. At 30 min, most of the peptide had agglomerated paranuclearly, with a few isolated aggregates in the nucleus. At 60 and 120 min, the peptide appeared to disperse and most of the fluorescent material had a diffuse localization around the nuclear area (Figure 8). These results revealed that CEMP1-p4 possessed several desired properties, such as solubility, rapid cell penetration and intracellular retention, and shows specific perinuclear and nuclear localization.

### 2.9. β-Catenin Translocation by CEMP1-p4

**Figure 9:**
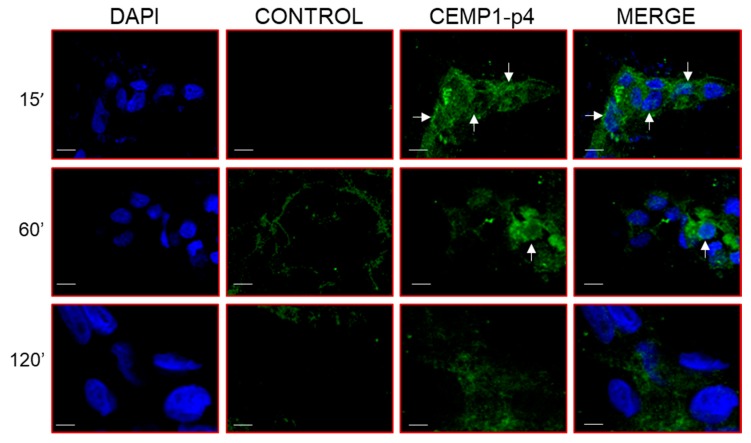
Translocation of *β*-catenin following HOMSC stimulation with CEMP1-p4 (4 µg/mL) was determined using antihuman *β*-catenin rabbit polyclonal antibody. Beta-catenin was localized in the cytoplasm from 15 up to 60 minutes. At 120 min after the treatment with CEMP1-p4, *β*-catenin began to be translocated into the nucleus. Image is representative of data from three independent experiments. Arrows indicate punctuate expression of *β*-catenin into the cell nucleus. Original magnification, 3400. Bar scale = 10 μm.

Our results showed that after 15 min in the HOMSCs treated with CEMP1-p4, *β*-catenin expression was located surrounding the nuclear membrane, into the nucleus and cell membrane in a punctuate manner (Figure 9). At 1 hr, *β*-catenin’s punctuate expression was more intense in the nucleus and cell cytoplasm. However, at 2 hrs after treatment, *β*-catenin almost disappeared from nucleus and was weakly located at the cell cytoplasm level (Figure 9). 

## 3. Discussion 

The findings of this study show that a synthetic 15 amino acid-long peptide (CEMP1-p4), corresponding to CEMP1′s *C*-terminus, activates *β*-catenin signaling in HOMSCs, inducing its differentiation to a “mineralizing-like” phenotype. This sequence shows 80% of intrinsic disorder-promoting amino acids in its composition. Therefore, it belongs to CEMP1′s most intrinsic disordered region. The plasticity of the protein’s *N*- and *C-*terminus regions show properties associated with crystal nucleation, growth and binding to hydroxyapatite crystals during the biomineralization process [20,21]. Our experimental data support this statement, since physicochemical characteristics of CEMP1-p4 revealed by circular dichroism showed that CEMP1-p4 has a random-coiled structure. DLS experimental data showed that CEMP1-p4 formed three different states of oligomerization—the monomer state with a hydrodynamic ratio of 0.5 nm, and two oligomers of 125 nm and 5.0 µm. DLS detected the peptide-peptide complex formation in a buffer solution, demonstrating for the first time a possible self-assembly ability.

CEMP1-p4 induces the formation of spheres with an amorphous calcium phosphate (ACP) center and has a significant effect on the mode of hydroxyapatite formation. The particles present in the center of the spheres eventually transformed to plate-like crystals, and due to its physicochemical properties, CEMP1-p4 induces hydroxyapatite crystal nucleation confirmed by EDS analyses that revealed a 1.52 Ca/P ratio. Acidic and disordered sequences accomplish several roles in crystal formation and controlling hydroxyapatite crystal growth. Indeed, peptides involved in biomineralization, such as CEMP1-p4, have a random-coil secondary structure that allows intermolecular interactions at the peptide-mineral interface [22,23], probably by the interaction of CEMP1-p4 with phosphate ions caused by conformational changes in CEMP1-p4 that would promote the transformation of the amorphous phase to the crystalline phase [24]. 

Recently, mesenchymal stem cells isolated from the lamina propria of human oral mucosa [25,26] have shown to be multipotent and are able to differentiate into multilineage tissues, including bone, cartilage, adipose tissue, Schwann-like cells and neurons in vitro and in vivo [25,27,28,29]. Here, this study showed that HOMSCs treated with CEMP1-p4 increased their proliferation to a larger extent when compared with 10% FBS. We found that CEMP1-p4 triggers the expression of *β*-catenin as early as 15 min at the mRNA and protein level by 6.6- and 2.0-fold when compared with controls, respectively. It is also showed that CEMP1-p4 inactivates GSK-3*β*, which increased the proliferation of HOMSCs, indicating that CEMP1-p4 possesses a growth factor-like activity. Because the *β*-catenin signaling pathway plays an important role during the mineralization process, we determined the expression of Gsk3b, Tcf1 and Lef1, which are fundamental to the Wnt/*β*-catenin signaling pathway. Our results showed that CEMP1-p4 enhanced the expression of *β*-catenin, while expression of Gsk3b decreased and downstream transcriptional factors Tcf1 and Lef1 were activated by CEMP1-p4 treatment [30], revealing that CEMP1-p4 activated the Wnt/*β*-catenin signaling pathway, inducing HOMSC differentiation to a “mineralizing-like” phenotype. More importantly, CEMP1-p4 appears to induce osteoprogenitor proliferation and differentiation by uncommitted HOMSCs. Gene expression at the mRNA and protein level of mineralization-related molecules further supports this premise [31]. At the mRNA level, RUNX2 was expressed at initial stages of mineralization. This transcriptional factor is of singular importance since it promotes the differentiation of osteoprogenitors to adopt a mature osteoblastic phenotype [32], meanwhile, OSX was expressed at the middle stages of mineralization. This transcriptional factor is associated with the regulation of bone formation [33]. Integrin-binding sialoprotein (IBSP) is a glycoprotein that plays important roles as a nucleator of hydroxyapatite crystals, cell attachment and cell differentiation [34,35,36]. CEMP1-p4 induced IBSP expression by HOMSCs at the middle stages of mineralization. OCN shows affinity for Ca^2+^ and regulates hydroxyapatite and participates in the late stages of mineralization [22,37,38]. Its expression implies the onset of mineralized matrix deposition. Significantly, at the protein level, CEMP1-p4 induces the strong expression of RUNX2 and OCN from 3 to 14 days when compared to controls. OSX was highly expressed at the initial and late stages of mineralization, and IBSP showed strong expression at the middle and late stages of mineralization. Increases in ALP activity by CEMP1-p4 and calcium nodule formation denotes that HOMSCs acquired a “mineralizing-like” phenotype [22,37,38]. These data taken together indicate that CEMP1-p4 leads HOMSCs to an osteoprogenitor cell phenotype through the Wnt/*β*-catenin pathway. The differentiation of osteoprogenitors is under the regulation of various signaling pathways, a number of transcriptional factors that fundamentally determine the fate of stem cells toward a “mineralizing-like” phenotype [39,40,41,42,43,44,45,46]. Active Wnt signaling has been demonstrated to be required for conserving the pluripotency and proliferation of embryonic stem cells [16,17], as well as stem cell fate determination [47,48]. Wnt/*β*-catenin is a principal pathway related to osteoblast differentiation [40,41,43,49,50,51,52,53], and it has been shown that in the presence of Wnt signaling, *β*-catenin accumulates in the cytoplasm and translocates into the nucleus, where it associates with Tcf/Lef transcription factors to regulate the expression of target genes [46,54,55,56,57]. *β*-catenin plays a direct role in osteoblast differentiation and some aspects of CEMP1-p4 may be mediated by *β*-catenin signaling [58,59,60]. However, *β*-catenin alone is not enough to induce osteogenic-like cell differentiation, unless other stimulatory signals are present [61]. We believe that one stimulatory factor is CEMP1-p4, since it promotes upregulation of RUNX2 expression [62]. This is supported by our findings, since β-catenin’s expression was located surrounding the nuclear membrane, in the nucleus and cell membrane as early as 15 min, and after 1 hr, *β*-catenin’s expression was more intense in the nucleus and cell cytoplasm. Our results indicate that CEMP1-p4 increases *β*-catenin expression, which make osteoprogenitor cells capable of differentiating to osteoblasts [39,63]

Peptides show a propensity to bind flat protein surfaces, however, most peptides are incapable of passing through the cell membrane and therefore, their usefulness is limited. Cell-penetrating peptides (CPPs) either contain mainly positively charged amino acids or an alternating pattern of charged and hydrophobic side chains [64,65]. Hydrophobicity is crucial for bilayer insertion and penetration, as hydrophobic amino acids interact with the core of membranes [66,67,68]. Using confocal microscopy, we examined the subcellular location of fluorescein-labelled CEMP1-p4. The peptide showed a perinuclear localization as early as 1 min after its addition to the medium. The CEMP1-p4 peptide organized into homogeneous aggregates, adopting a paranuclear localization and a few aggregates in the nucleus. As the time advanced, CEMP1-p4 accumulated into vesicular structures, possibly endosomes [69]. Some characteristics are necessary for efficient cell uptake of CPPs: a) hydrophobicity to a certain degree; and b) stretches of hydrophobic and cationic amino acids. Cemp1-p4 possesses these characteristics and could be the reason why the peptide penetrated the cell membrane and adopted a paranuclear location only one min after its addition. This is crucial for the activation of the Wnt pathway [70,71,72,73] and of clinical relevance, since bioactive and cell-permeable peptides represent a promising strategy for the application of peptide-derived drugs. Our findings of *β*-catenin signaling in HOMSCs and its stimulation by CEMP1-p4 might have potential therapeutic implications for the repair and regeneration of mineralized tissues including bone, cementum and dentin. 

## 4. Materials and Methods

### 4.1. Peptide Synthesis

A sequence corresponding to human cementum protein 1 (CEMP1; Accession: NP_001041677.1; GI: 115292438), *C*-terminal domain with an amino acid sequence QGQGDTEDGRMTLMG (CEMP1-p4); corresponding to 233–247 aa was synthesized by solid phase techniques (Fmoc). Then, C-18 reversed-phase liquid chromatography purification was performed up to 95% (New England Peptide, Ipswich, MA, USA). The lyophilized peptide was dissolved in distilled deionized water and filtered (0.22 mm filter) before use.

### 4.2. Characterization of CEMP1-p4 Synthetic Peptide

To determine if CEMP1-p4 possessed a secondary structure, the peptide was analyzed by circular dichroism spectroscopy (CD) and its behavior in solution was analyzed by dynamic light scattering (DLS). 

### 4.3. Circular Dichroism

Synthetic CEMP1-p4 was dissolved in 10 mM sodium phosphate buffer, pH 7.4 at 200 μg/mL concentration. CD spectra were collected using a JASCO J-710 spectropolarimeter (cell length = 1 mm). CD data were collected from 190–260 nm. A baseline 10 mM sodium phosphate, pH 7.4 buffer solution was recorded separately and subtracted from each spectrum. CD data were analyzed by the program Dicroprot to determine CEMP1-p4′ secondary structure with the Henessey & Johnson algorithm [74].

### 4.4. Dynamic Light Scattering

Measurements of the CEMP1-p4 solution were acquired with a Zetasizer Nano S (Malvern Instruments, Ltd., Clevedon Organs, UK) molecular sizing instrument [75]. The temperature was held at 37 (0.1 °C) with a Peltier unit. Data analyses were performed using the software Zetasizer Nano Family age (V.7.10), (Malvern Instruments, Ltd., Clevedon Organs, UK). 

In silico analyses—the isoelectric point (pI), molecular weight, hydrophobic character and GRAVY of the CEMP1-p4 peptide were determined by using the ExPASy tool ProtParam. (https://web.expasy.org/protparam/).

### 4.5. Mineralization in a Cell-Free System

Calcium phosphate crystal formation was induced by CEMP1-p4 in a cell-free system, which consisted of a semisolid medium at physiological pH at RT (room temperature) as described elsewhere (Montoya et al. 2014). Briefly, a sodium metasilicate (SMS) solution of specific gravity 1.06 g/mL, 1 MH3PO4, HEPES buffer 10 mM, pH 7.2 was used. CEMP1-p4 peptide (10 μg/mL) was dissolved in 10 mM HEPES pH 7.4 and mixed with the SMS. After gel formation, 100 mM CaCl_2_ in 10 mM HEPES buffer, pH 7.4, was added over the crystallizing medium. Gels were incubated at 37 °C for 7 days. Bovine serum albumin (20 µg/mL) was used as a negative control. Elemental chemical composition and morphology of the crystals were performed by scanning electron microscopy (JSM 5600LV; JEOL USA Inc., Peabody, MA, USA). The calcium to phosphate ratio (Ca/P) was obtained by the energy-dispersive X-ray spectroscopy (EDS) analysis (Noran X-ray microanalysis detector, model Voyager 4.2.3; Thermo Scientific, Waltham, MA, USA). Once the composition was obtained, the crystals were covered with a 100 nm thick gold film.

### 4.6. Cell Culture

HOMSCS were isolated and grown as described elsewhere [6]. Human tissue from the oral mucosa was obtained from donors that underwent routine oral surgery procedures. The protocol was reviewed and approved by the Ethics Committee at the National University of Mexico School of Dentistry (UNAM), 9 March 2018; Register # CIE/0303/02/2018. For the experiment, cells between the 2^nd^ and 5^th^ passages were used. HOMSCs were maintained in DMEM supplemented with 10% fetal bovine serum (FBS; Thermo Fisher Scientific, Waltham, MA, USA), and antibiotics (penicillin 10 UI/mL, streptomycin: 100 µg/mL), and cultured in 5% CO^2^ and 95% air atmosphere with 100% humidity. Mineralizing media (MM), consisting of materials described above plus 5 mM *β*-glycerophosphate, ascorbic acid (50 µg/mL) and dexamethasone (10^−7^ M), was used.

### 4.7. Cell Proliferation Assay

The effect of CEMP1-p4 on proliferation was performed by using the colorimetric MTT assay 3-(4,5-dimethylthazol-2-yl)-2,5-diphenyl tetrazolium bromide (Boehringer Mannheim, Indianapolis, IN, USA). HOMSCs were plated at 0.5 × 10^4^ density in 96-well plates (Thermo Fisher Scientific). After overnight attachment, experimental cells were treated from 0 up to 96 h with several CEMP1-p4 concentrations (0, 2, 4, 6, 8 and 10 µg/mL) in medium containing 0.2% FBS. Control cells without CEMP-1-p4 treatment were cultured in the presence of 10% FBS (positive control), or in presence of 0.2% FBS alone (negative control). At the end of the treatment intervals (every 24 h), MTT solution (5 mg/mL) in PBS was added to the wells and incubated at 37 °C for 4 h. Lysing buffer (20% sodium dodecyl sulfate, 50% dimethyl formamide (pH 4.7) was added to each well and plates were incubated for 1 h at 37 °C. The resultant solution was read at 570 nm on a microplate reader (Filter Max F5; Molecular Devices, Sunnyvale, CA, USA). 

### 4.8. Time Course Experiments

To identify the signal transduction pathways involved in the regulation of gene expression by CEMP1-p4 at the mRNA and protein levels, HOMSCs cells were plated at 5 × 10^4^ density in 6-well culture plates and culture conditions were as follows—1) 0.2% FBS plus 4.0 µg/mL of CEMP1-p4 (experimental), and 0.2% FBS alone (negative control) and 10% FBS (positive control). The CEMP1-p4 dose was selected according to the previous findings from the dose-response on cell proliferation. Total RNA and proteins were extracted at 0′, 15′, 30′, 60′ and 120′ after treatment. At the mRNA and protein level, the expression of *β*-catenin, Gsk3b, Tcf1 and Lef1 was performed.

### 4.9. HOMSC Mineralization

In order to determine the effect of CEMP1-p4 on the induction of the mineralization process in vitro, HOMSCs were plated at 5 × 10^4^ density in 6-well plates (Thermo Fisher Scientific, CDMX, Mexico) and allowed to attach overnight. Cells were cultured for 5, 10 and 15 days under the following conditions—10% FBS plus 3 mM *β*-glycerophosphate and 50 mg/mL ascorbic acid (controls) or with 4 µg/mL of CEMP1-p4 (experimental).

### 4.10. Alkaline Phosphatase Activity

HOMSCs were plated at 5 × 10^4^ density in 6-well culture plates and cultured for 5, 10 and 15 days in the conditions as previously described [12]. Alkaline phosphatase (ALP)-specific activity was performed as described by Lowry et al. [76]. The activity was expressed as nanomoles of p-nitrophenol per minute per milligram of protein. Protein content was determined as described elsewhere [77]. 

### 4.11. Alizarin Red Staining and Quantification of Mineralization 

HOMSCs were plated at a density of 5 × 10^4^ cells, in 6-well plates (Thermo Fisher Scientific) in the conditions described above. Culture medium was replaced every other day and cells were fixed with 4% paraformaldehyde and rinsed with PBS. Cultures were stained with 2% Alizarin Red S at pH 4.2 (ARS; Millipore Sigma, Burlington, MA, USA) for 10 min. Calcium deposits were documented and analyzed with ImageJ software (National Institutes of Health (NIH), Bethesda, MD, USA) Mineralization was quantified by the cetylpyridinium chloride (CPC) method [12]. Briefly, alizarin red was diluted in a 2 mL/well with 10% CPC, and incubated for 1 h at 37 °C. Then, the solution was diluted five times in CPC, and the absorbance reading was at 570 nm (Filter Max F5; Molecular Devices, LLC. San Jose, CA, USA). Experiments were performed in triplicate and repeated twice.

### 4.12. Real-Time Quantitative RT-PCR

HOMSCs were cultured under conditions as described above for 5, 10, and 15 d. Cells were collected and total RNA was extracted with Trizol Reagent (Thermo Fisher Scientific, Waltham, MA, USA). Ten RNA nanograms were used per reaction, and the level of mRNA expression was quantified by the one-step real-time quantitative RT-PCR (RT-qPCR) method using Super-Script III Platinum Sybr Green One-Step qPCR Kit (Thermo Fisher Scientific). The conditions for a 25 µL reaction were as follows—cDNA synthesis at 50 °C for 3 min, denaturation at 95 °C for 5 min; thereafter 40 cycles of 95 °C for 15 s, 60 °C for 30 s, and a 40 °C hold cycle for 1 min. Reactions were performed in a Corbett Rotor-Gene 6000 (Qiagen, Germantown, MD, USA). Primer sequences for human genes encoding IBSP, OCN, runt-related transcription factor-2 (RUNX)-2, osterix (OSX), *β*-catenin, Gsk3b, Tcf1, Lef1 and glyceraldehyde 3-phosphatedehydrogenase (GAPDH) are shown in Table 1.

### 4.13. Western Blot Analysis

HOMSCs were plated at 5 × 10^4^ density in 6-well culture plates. Culture conditions were as described above and cells cultured for 5, 10, and 15 d. At the end of each term, the cells were scraped with a policeman and dissolved in lysis buffer containing 1% SDS and a protease inhibitor cocktail [7]. Western blots were performed with rabbit polyclonal antibodies against human OSX, RUNX2, OCN, CEMP1, CAP, GAPDH, and a monoclonal antibody against human IBSP, (Santa Cruz Biotechnology, Dallas, TX, USA), WNT3*α*, GSK-3*β*, *β*-catenin, Tcf and Lef1 (Jackson Immuno Research Lab, Inc. West Grove, PA, USA). Proteins from experimental and control groups (20 mg/lane) were separated on a 12% SDS-PAGE and electroblotted onto a PVDF membrane (Immobilon-P; Millipore Sigma). Membranes were blocked with 5% nonfat milk for 1 h and then incubated with 1:1000 diluted antibodies for 1 h, then washed with PBS three times. After they were washed, the membranes were incubated with 1:1000 diluted horseradish peroxidase-conjugated goat antirabbit, or goat antimouse IgG secondary antibodies for 1 h. Membranes were washed with PBS and developed as described elsewhere [7]. For assessment of the Western blot, all membranes were digitalized with an Epson V550 scanner and processed and analyzed with ImageJ software (National Institutes of Health (NIH), Bethesda, MD, USA). The relative level of each protein was assessed by measuring the integrated intensity of all pixels in each band, excluding the local background. Results are expressed as percentages of protein intensity.

### 4.14. Confocal Microscopy Analysis 

In order to determine CEMP1-p4 internalization and location, HOMSCs were plated at 0.5 × 10^3^ in Lab-Tek chamber slides. Cells were cultured overnight and incubated for 1, 15, 30, 60 and 120 min with CEMP1-p4 labelled with Alexa Fluor 546, as described elsewhere [24]. Fluorescence images were obtained using an Olympus FV1000 *c*onfocal laser scanning microscope (Olympus America, CDMX, México) fitted with a ×20 objective lens. Control cells were incubated with Alexa 546 alone. Images were analyzed using the plot profile option in ImageJ.

### 4.15. Immunofluorescence

HOMSCs treated with CEMP1-p4 at 4 µg/mL were incubated for 15, 60 and 120 min, then fixed in 4% paraformaldehyde for 10 min at RT, incubated overnight at 4 °C with a Moab to *β*-catenin (BD Biosciences, San Jose, CA, USA). For nuclear localization analysis, the fixed samples were subjected to fluorescent digital confocal imaging analysis using a Zeiss LSM 510 confocal microscope (Carl Zeiss, CDMX, México).

## 5. Conclusions

This study shows for the first time the involvement of the Wnt/*β*-catenin signaling pathway in CEMP1-p4-mediated differentiation in HOMSCs. The role of a CEMP1-p4 suggests that the intracellular mechanism associated with the activation of trigger genes to induce stem cell differentiation towards an osteoblastic phenotype is through the activity of the molecules of the canonical Wnt/*β*-catenin signaling pathway induced by CEMP1-p4. However, it is still necessary to know the mechanism of interaction of this peptide with these molecules that allow for recognition if it is through interactions by phosphorylation that allow for activating or inactivating of molecular complexes associated with this pathway.

## Figures and Tables

**Table 1:** Primers Used for RT-qPCR.

Gene	Primer Sequence 5´-3´	
	Forward	Reverse
*β-CATENIN*	GGTGCTGACTATCCAGTTG	GGCAGAGTAAAGTATTCACCC
*GSK3* *β*	GACTTTGGAAGTGCAAAGC	AGGAAATATTGGTTGTCCTAGC
*LEF1*	GAAGAGGAGGGCGACTTAG	CTTTCCTTCATCAGGGTGTTC
*TCF1*	CCAACATTCTCAGGTCGC	GAGCAAGCCAGGTGTTC
*IBSP*	AACGAAGAAAGCGAAGCAGAA	TCTGCCTCTGTGCTGTTGGT
*OCN*	GTTGCAGGCTCAATCCATTT	CCATCCTCATACCTGCACCT
*OSX*	GCCAGAAGCTGTGAAACCTC	GCTGCAAGCTCTCCATAACC
*RUNX2*	ACCCAGAAGGCACAGACAGAAG	AGGAATCGCCCCTAAATCACT
*GAPDH*	CAACGGATTTGGTCGTATTGG	GCAACAATATCCACTTTACCAGAGTTAA

## Abbreviations

CAP	Cementum Attachment Protein
CEMP1	Cementum Protein 1
CEMP1-p4	Cementum Protein1-Peptide 4
CD	Circular Dichroism
CPC	Cetylpyridinium Chloride
CPPs	Cell Penetrating Peptides
DMEM	Dulbecco’s Modified Eagle Medium
DLS	Dynamic Light Scattering
EDS	Energy Dispersive X ray spectroscopy
FBS	Fetal Bovine Serum
Fmoc	Fluorenylmethyloxycarbonyl
GAPDH	Glyceraldehyde-3-Phosphate Dehydrogenase
GRAVY	Grand Average of Hydropathicity
GSK3β	Glycogen synthase kinase 3 beta
HA	Hydroxyapatite
HEPES	2-[4-(2-hydroxyethyl) piperazin-1-yl] ethanesulfonic acid
HOMSCs	Human Oral Mucosa Stem Cells
IBSP	Integrin Bone Sialoprotein
IDP	Intrinsically Disordered Protein
LEF1	Lymphoid Enhancer binding Factor 1
MTT	3-(4,5-dimethylthiazol-2-yl)-2,5-diphenyltetrazolium bromide
OCN	Osteocalcin
OSX	Osterix
RUNX2	Core-Binding Factor Subunit Alpha-1
RPLC	Reverse-Phase Liquid Chromatography
SEM	Scanning Electron Microscopy
SMS	Sodium Metasilicate
TCF	T-cell transcription Factor
Wnt	Wingless-related integration site
